# Free Sugars Consumption and Type 2 Diabetes: What Are the Concerns and How Strong is the Evidence?

**DOI:** 10.1007/s13668-026-00740-w

**Published:** 2026-02-13

**Authors:** Jimmy Chun Yu Louie, Sze-Yen Tan

**Affiliations:** 1 Discipline of Dietetics, Department of Allied Health, School of Health Sciences, Swinburne University of Technology, Swinburne Place West Building, 1 John St, Hawthorn, VIC 3122 Australia; 2 Institute for Physical Activity and Nutrition, School of Nutrition and Exercise Sciences, Deakin University, Geelong, VIC Australia

**Keywords:** Free sugars, Added sugars, Type 2 diabetes, Sugar-sweetened beverages

## Abstract

**Purpose of Review:**

This review examines the relationship between free sugars consumption and type 2 diabetes mellitus (T2DM) risk, with particular focus on the differential effects of sugar types and their delivery forms. Given the unresolved questions and areas of uncertainty in the literature, this analysis aims to clarify the evidence base and inform public health and clinical strategies.

**Recent Findings:**

Epidemiological studies and meta-analyses show inconsistent associations between total sugar intake and T2DM risk. Sugar-sweetened beverages (SSBs), however, are more consistently linked to increased risk, with estimates indicating a 13–30% rise in T2DM risk per daily serving. In contrast, free sugars consumed in solid foods, at typical dietary levels, appear to be effectively metabolized without clear harmful effects for T2DM risks. Experimental evidence indicates that the small intestine plays a key role in metabolizing fructose before it reaches the liver, limiting its contribution to hepatic lipogenesis and associated metabolic disruptions—provided intake remains within normal dietary ranges. Proposed mechanisms for the stronger association with SSBs include faster absorption, minimal satiety response, and reduced dietary compensation, which may contribute to overall excess energy intake. Measurement challenges and heterogeneity across studies complicate interpretation.

**Summary:**

The form in which sugar is consumed appears more relevant to T2DM risk than the type of sugar itself. Evidence supports focusing public health efforts for T2DM prevention on reducing SSB consumption rather than targeting total free sugar intake. Future research should explore dose-response effects, long-term outcomes, and how individual metabolic profiles interact with different sugar sources.

## Introduction

Type 2 diabetes mellitus (T2DM) is a global health crisis, with the International Diabetes Federation estimating that 537 million adults were living with diabetes globally in 2021, a number projected to rise to 783 million by 2045 [1]. Most of these cases are T2DM, characterized by insulin resistance and progressive *β*-cell dysfunction. The rising prevalence of T2DM is a significant public health concern, as the disease is associated with disability and a range of debilitating complications, including cardiovascular disease, kidney disease, nerve damage, and premature death [1].

Free sugars, defined by the World Health Organization as “*all monosaccharides and disaccharides added to foods by the manufacturer*,* cook*,* or consumer*,* plus sugars naturally present in honey*,* syrups*,* and fruit juices*” [2], have become a focal point in nutritional research and practice and public health policy. A closely related concept is “added sugars”, used in U.S. Dietary Guidelines [3], which encompasses sugars added during processing or preparation but excludes those naturally present in fruit juices [4, 5]. While conceptually similar, these definitions differ in their treatment of fruit juice sugars, a distinction with implications for dietary guidance. Throughout this review, we use “free sugars” as the primary framework while noting where the added sugars definition may yield different interpretations.

The increased focus on free sugars is largely due to evidence of their role in the development of various non-communicable diseases (NCDs), including T2DM. In the context of public health, the main free sugars typically consumed by individuals include sucrose (a disaccharide made up of 1 each of glucose and fructose), high fructose corn syrup (HFCS, typically used in the U.S.), and fruit juices and their concentrates. It is important to note that while the name of HFCS implies a high fructose content, the main types of HFCS used in the food supply typically contain 45–55% by weight as fructose (with the rest being glucose), which is somewhat similar to the 50:50 proportion of the two monosaccharides found in sucrose [6].

The relationship between free sugars consumption and T2DM has been extensively examined [7–9], with substantial consensus emerging on several key aspects, particularly regarding sugar-sweetened beverages (SSB). Building upon comprehensive prior work [6, 10–12], and comprehensive assessments by European Food Safety Agency (EFSA) [13], which have thoroughly established the foundational evidence on sugar metabolism and health outcomes, this narrative review advances the field by integrating mechanistic understanding with practical implementation challenges. Specifically, we examine how different sugar sources should be treated in dietary guidance, critically evaluate metabolic effects at physiologically relevant doses (25–50 g fructose per meal, representing 1–2 SSBs or typical meal-associated sugar intake), and identify remaining evidence gaps to inform future research and policy development.

## Methods

This narrative review synthesizes current evidence on the relationship between free sugars consumption and T2DM risk, published between January 2000 and November 2025. The narrative format was deliberately chosen to integrate mechanistic understanding with epidemiological findings and provide critical analysis of how evidence translates into dietary guidance, rather than conducting a comprehensive systematic search with predetermined outcomes. Literature was identified through PubMed/MEDLINE searches, supplemented by reference list examination of key papers and recent systematic reviews. We prioritized studies that specifically addressed free sugars in different delivery forms, particularly comparing SSBs to solid food sources, with special attention to large prospective cohort studies, meta-analyses, and mechanistic studies elucidating physiological pathways linking sugar consumption to diabetes risk. For observational studies, we prioritized prospective cohort designs over cross-sectional studies, while for intervention studies we focused on randomized controlled trials with appropriate control conditions. Papers were selected based on their relevance to understanding the relationship between sugar consumption patterns and T2DM risk, with particular emphasis on studies that controlled for confounding factors and considered broader dietary patterns.

## Physiological Mechanisms: Types and Dose of Sugars

**Fig. 1:**
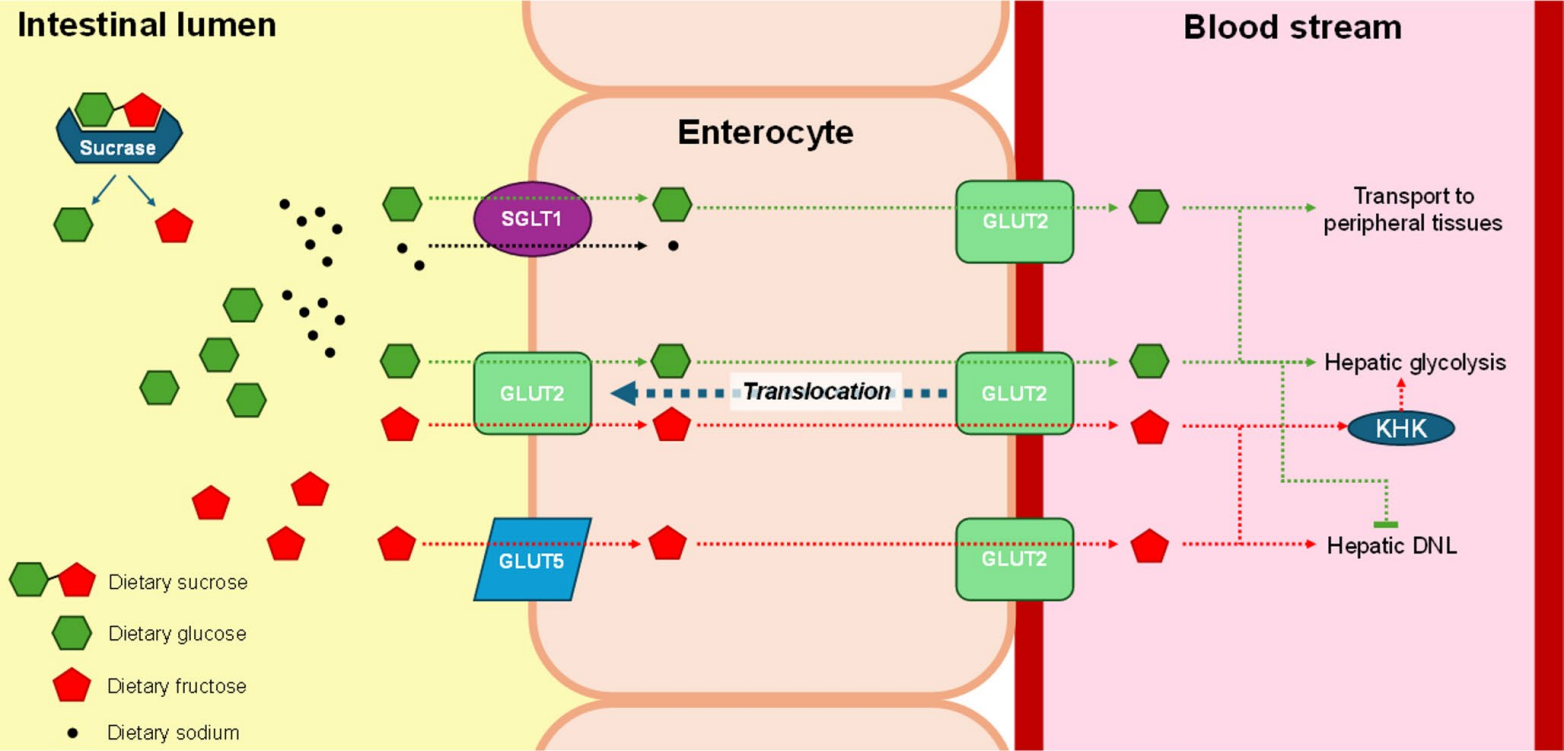
Absorption pathways of fructose and glucose in the small intestine. DNL, *de novo* lipogenesis; GLUT2, glucose transporter 2; GLUT5, glucose transporter 5; KHK, ketohexokinase; SGLT1, sodium-dependent glucose transporter 1

**Fig. 2:**
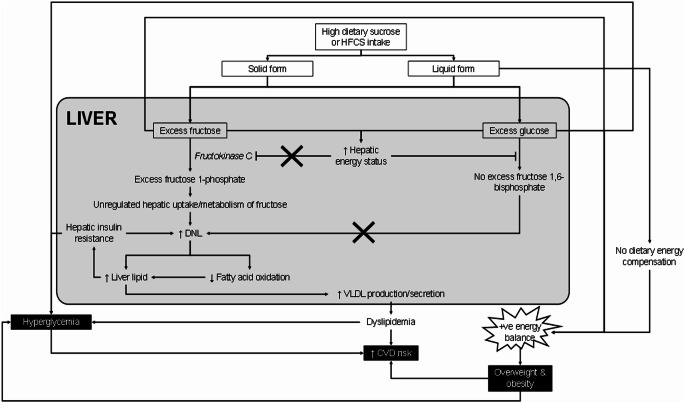
The proposed mechanism(s) of the adverse effect of high fructose consumption on metabolic health. CVD, cardiovascular disease; DNL, *de novo* lipogenesis; HFCS, high-fructose corn syrup; VLDL, very-low-density lipoprotein

The metabolic pathways for sugar absorption and metabolism are well-established [14–19]. Sucrose is hydrolysed in the small intestine, releasing glucose and fructose that are absorbed through distinct transporters (SGLT1 and GLUT2 for glucose; GLUT5 for fructose) (Fig. 1). While glucose transport is tightly regulated through insulin-mediated mechanisms, fructose follows a less regulated pathway to the liver, where it undergoes metabolism that can contribute to DNL and hepatic lipid accumulation when consumed in an extreme single dose (> 75 g fructose, equivalent to > 3 SSBs consumed in one sitting) (Fig. 2). These differential metabolic fates have led to proposals that fructose uniquely contributes to insulin resistance and T2DM development through mechanisms including increased hepatic lipogenesis and *β*-cell glucotoxicity [14–18].

However, this “fructose-specific” hypothesis faces important logical inconsistencies [19].

The glucose component of sucrose or HFCS undergoes identical metabolic pathways as glucose coming from other digestible carbohydrates (e.g., starch). Furthermore, glucose serves as the primary and essential energy source for the brain and many other tissues, with the central nervous system requiring approximately 120 g of glucose daily for normal function [20]. The body maintains tight homeostatic control over blood glucose concentrations precisely because of this metabolic essentiality. If the glucose portion of sugars were problematic, other carbohydrate sources such as starch, which is predominantly digested into and absorbed as glucose, should warrant equivalent concern. Conversely, if sugars are uniquely harmful, the effect would logically be attributable only to fructose.

Yet this creates a paradox. Fructose is not an essential nutrient and must be converted to glucose, lactate, or other metabolic intermediates before serving broader physiological functions [11]. This lack of essentiality, combined with its less tightly regulated hepatic metabolism, has placed fructose under particular scrutiny regarding potential adverse metabolic effects [21, 22]. However, fructose molecules are chemically identical regardless of source, meaning fructose from fruits and vegetables should pose equivalent risks to that from added sugars. Since fruits and vegetables are consistently recommended in dietary guidelines worldwide [23, 24], this paradox suggests that focusing solely on sugar type (glucose vs. fructose) oversimplifies a more complex issue.

A critical but often overlooked consideration is the differential effect of typical vs. extreme dosages [14, 25]. At physiologically relevant meal intake levels (25–50 g fructose), both glucose and fructose are efficiently metabolized without overwhelming regulatory capacity. The liver’s ability to process fructose at typical dietary doses results in relatively modest increases in lipogenesis and hepatic lipid accumulation [19]. In contrast, at extreme single doses exceeding 75 g fructose as a bolus, intestinal absorption mechanisms themselves become saturated [26, 27]. This saturation leads to incomplete fructose absorption from the intestinal lumen, with unabsorbed fructose entering the colon where it can cause gastrointestinal symptoms including bloating and diarrhea [27]. Such absorption ceiling creates a physiological plateau effect on hepatic DNL and intrahepatic lipid accumulation, as fructose that is not absorbed cannot contribute to these metabolic processes. This dose-dependency suggests that the specific type of sugar may not be the primary factor linking free sugar intake to T2DM risk. Instead, the delivery form of sugars warrants closer examination [23, 24].

## Influence of Sugar Forms

Recent umbrella reviews provide comprehensive syntheses of the evidence linking sugar intake to T2DM risk. Banjarnahor, et al. [28] systematically reviewed the corpus of systematic reviews on this topic, while Della Corte, et al. [29] conducted an updated meta-analysis of prospective cohort studies examining various sugar sources and T2DM incidence. These analyses consistently demonstrate that while total sugar or fructose intake shows no clear association with T2DM risk, a robust relationship emerges specifically for SSBs [30–34]. The strength of evidence for SSBs contrasts sharply with the heterogeneous and largely *null* findings for total sugars, suggesting that delivery form rather than sugar type per se drives the T2DM association. Notably, the SSB-T2DM relationship persists after adjustment for body mass index, indicating that the association operates through mechanisms beyond simple adiposity-mediated pathways.

When a large bolus of sugars (> 50 g fructose) is consumed rapidly in a readily absorbed form, such as from an SSB, this leads to a rapid increase in blood glucose and fructose levels [35]. This rapid influx exceeds the rate at which hepatic fructose metabolism and glucose-stimulated insulin secretion can maintain homeostasis, resulting in prolonged postprandial hyperglycaemia and increased hepatic DNL [36]. Animal studies demonstrate that liquid sugar intake triggers significantly enhanced expression of intestinal sugar transporters (GLUT2, GLUT5) and cholecystokinin in the ileum, suggesting adaptive upregulation in response to increased sugar exposure [18, 37, 38]. These findings are corroborated by human studies showing elevated GLUT2 and GLUT5 expression in obese compared to lean individuals, indicating potential transporter modulation in obesity [38].

Beyond absorption kinetics, SSBs contribute to T2DM risk through multiple additional pathways. The moderate-to-high glycaemic index of SSBs creates substantial glycaemic load [39], which may exceed compensatory insulin secretory capacity, particularly in individuals with metabolic syndrome or insulin resistance, thereby promoting hyperinsulinemia and sustained hyperglycaemia. Furthermore, fructose metabolism in SSBs generates hepatic uric acid through the DNL pathway, contributing to oxidative stress, inflammation, and potential inhibition of nitric oxide production which are factors that further impair insulin sensitivity [40].

In contrast, when sugars are consumed in solid forms such as whole fruits, the digestion and absorption are slowed by the food matrix, including fibre content, leading to a more gradual release of sugars into the bloodstream [38]. Intact whole fruits consistently demonstrate lower glycemic responses and greater satiety effects compared to the same fruits when juiced [41] (but not pureed [42]). This slower absorption allows hepatic metabolism and insulin secretion to process sugars more effectively, minimizing the risk of metabolic perturbations. Additionally, the fibre and nutrient content of whole fruits enhances satiation, promoting more complete compensatory reductions in subsequent energy intake compared to the incomplete compensation observed with liquid calories [43]. The difference in glycaemic response and satiety between whole and processed fruit is attributable to the disruption of cellular structure during juicing, which increases the rate of sugar release and absorption, and the removal of fibre, vitamins and associated matrix effects that slow gastric emptying and glucose absorption [44, 45]. The critical distinction, therefore, lies not just in the total amount of sugars consumed, but in their delivery form and the rate at which they enter systemic circulation.

It is important to recognize that SSB consumption clusters with other dietary and socioeconomic risk factors for cardiometabolic disease. Population studies consistently demonstrate that higher SSB consumption is associated with lower socioeconomic status, including reduced income, net worth, and educational attainment, which are factors independently linked to increased T2DM risk [46–48]. Additionally, SSB consumers tend to have lower overall healthy eating index scores and higher consumption of fast food and other ultra-processed foods [48, 49], creating a constellation of dietary risk factors that may act synergistically to increase T2DM risk beyond the direct metabolic effects of sugar intake alone. Importantly, the confounding factors that may inflate SSB-T2DM associations likely operate in the opposite direction for fruit juice, as fruit juice consumption is associated with higher socioeconomic status, health-consciousness, and overall diet quality. This positive confounding could mask true harmful associations with fruit juice, contributing to the weaker epidemiological signals observed for this beverage category despite mechanistic similarities with SSBs regarding free sugar delivery.

Apart from the form of consumption, the metabolic fate of consumed sugars, particularly fructose, appears to also depend on the pattern of consumption. A recent animal study [50] using isotopic tracer of fructose showed that at up to 1 g per kg body weight (equivalent to approximately 70 g for a 70 kg person, or about 3 standard SSBs), fructose consumed in a bolus is effectively metabolized by the small intestine, with little making its way to the liver and the circulation. This intestinal fructose metabolism has also been shown in another animal study to shield the liver from fructose-induced steatosis [51]. While this suggests that fructose consumed throughout the day in moderate amounts (25–50 g per meal, or 75–150 g/day total from 3 meals) might have minimal hepatic impact, experimental evidence indicates that chronic consumption of liquid sugars at high intake levels (> 125 g/day free sugars, representing > 25% energy intake) may override these protective mechanisms. Both liquid high-sucrose and high-fructose diets resulted in enhanced liver triglyceride accumulation compared to controls, with liquid forms showing more pronounced effects than solid forms. These findings challenge the traditional view that dietary fructose per se is inherently hepatotoxic [22, 52–54], suggesting instead that the form of delivery may be the critical factor in determining metabolic outcomes.

It is important to note that the body’s capacity to maintain glucose homeostasis depends critically on the pattern and magnitude of sugar intake [55]. While rapid sugar consumption from SSBs transiently exceeds hepatic fructose metabolism capacity and insulin secretory response, producing acute postprandial effects including elevated hepatic DNL, these acute perturbations do not necessarily compromise overall glucose regulation when habitual free sugar intake remains moderate (35–50 g/day, representing the Western population median) [56]. The key distinction is between transient metabolic stress during individual postprandial periods and the body’s broader compensatory capacity to restore euglycemia. When free sugar intake is moderate and consumed across typical meal occasions rather than in concentrated doses, compensatory mechanisms effectively prevent progression to chronic hyperglycaemia or metabolic dysfunction.

The critical threshold is crossed when sugar intake becomes consistently excessive (free sugars > 125 g/day or > 25% of total energy, characterizing approximately 20–25% of adults in the US and UK), particularly from rapidly absorbed liquid sources. Under these conditions, the cumulative burden of repeated acute metabolic stress eventually impairs compensatory mechanisms, contributing to insulin resistance and T2DM development. This progression is especially likely with high SSB consumption, where consumption frequency often exceeds typical eating occasions, as most individuals consume beverages multiple times daily while maintaining relatively fixed meal patterns [57]. Compounding this frequency effect, liquids provide poor satiety signalling compared to solid foods [58], resulting in incomplete dietary compensation at subsequent meals [59]. It bears noting that saturation of intestinal monosaccharide absorption mechanisms would require consumption of approximately four SSB servings (approximately 75–80 g fructose as a bolus) within a short time frame, which does not represent typical consumption patterns (median of 1–2 SSBs/day among SSB consumers) for most individuals. Rather, the metabolic risk from SSBs stems primarily from their contribution to cumulative sugar exposure through frequent consumption combined with inadequate compensatory reduction in energy intake from other sources.

To date, the relationship between added/free sugar intake and T2DM has predominantly been examined with a focus on simple sugars that add sweetness to foods and beverages. Sweet taste is perceived as pleasant within a certain range of sugar concentrations [60], so, in other words, sugars can enhance the palatability of foods, and taste is often cited as an important factor that influences foods choice and consumption [61]. Although the influence of sweet taste liking on dietary intake is inconclusive largely due to heterogeneity in study design and methodology [61], there is some evidence that those who were classified as sweet-likers preferred higher concentration of sucrose, had more refined and total sugars, had higher frequency of sweet food consumption [62], and consumed more SSB [63, 64]. Therefore, one argument put forward by advocates for sugar reduction is that our liking of foods with added sugars may promote overconsumption, and result in weight gain and eventually obesity and its associated comorbidities, albeit this hypothetical link has recently been shown to be untrue [65]. While this is plausible, emerging evidence suggests that sugars consumed in a liquid form, such as those found in SSBs, and often-time as the only energy-yielding nutrient, have a more pronounced impact on promoting overeating compared to solid sugars [36, 66–68]. As summarized above, this effect is partly attributed to the high frequency and rate of consumption, and weak appetitive effects of beverages compared to solid foods. In solid foods, sugars are embedded in a matrix of fibers, proteins, and fats, and they are digested and absorbed more slowly [36]. This slower digestion, gastric emptying, and absorption process may contribute to enhanced satiation and satiety, and better appetite control and food intake regulation [69].

Moreover, the satiety response to liquid sugars is typically less robust than that to solid sugars [36, 59]. This distinction stems from the physiological response to liquid calories: when sugars are consumed in liquid form, they trigger less robust gastrointestinal satiety signals due to faster gastric emptying, resulting in reduced gastric distension and attenuated release of key appetite-regulating hormones, including insulin, glucagon-like peptide-1 (GLP-1), gastric inhibitory polypeptide (GIP), cholecystokinin (CCK), and peptide YY (PYY) [70]. While solid sugar consumption typically leads to compensatory reductions in subsequent food intake [36, 59], liquid sugars fail to prompt similar adjustments, which helps explain why high SSB consumption is associated with increased total energy intake and obesity risk. Although both forms of sugar can contribute to excessive caloric intake, the rapid absorption and inadequate satiety signaling associated with liquid sugars make them particularly problematic, providing strong evidence for why dietary guidelines should emphasize limiting SSB intake specifically.

## The Plausibility of other Mechanisms

While gut microbiota have been proposed as potential mediators, the rapid and efficient absorption of free sugars in the proximal small intestine suggests that direct metabolic effects are likely more important than microbiota-mediated mechanisms for typical intake levels (25–50 g fructose per meal, or 35–50 g/day total free sugars representing Western population median intake) [13, 71]. At typical doses, most free sugars are absorbed and metabolized effectively in the upper gastrointestinal tract, with little spilling over to the lower gut where they could influence the microbiome. Microbiota may play a role only at extreme doses (> 75 g fructose as a bolus, equivalent to > 3 SSBs consumed simultaneously) where fructose malabsorption occurs and sugars reach the colon [72, 73]. Also, the gut microbiome is a dynamic ecosystem, and the dysbiotic effects observed at higher consumption levels may not be as pronounced when free sugars are consumed in physiological amounts [73]. More studies are needed to verify if this mechanism explains the relationships between free sugar intake and T2DM.

Studies have also indicated that free sugars consumption may induce epigenetic changes [74], such as DNA methylation and histone modifications, which can impact gene expression and influence metabolic pathways involved in the pathogenesis of T2DM [75]. Excess sugar intake will result in excess production of acetyl-CoA, which acts as a substrate for histone acetyltransferases that is involved in histone modifications [76], and S-adenosylmethionine (SAM) which serves as a universal methyl donor in the methylation of DNA [77]. However, since acetyl-CoA and SAM are both intermediate metabolites of energy metabolism [78], their increase is directly caused by an excess substrate in the relevant biochemical pathway (i.e., glycolysis and TCA cycle) which is not unique to sugars – as mentioned above all digestible carbohydrates are metabolized using the same pathway. What remains unclear is whether different carbohydrate sources, while sharing common metabolic pathways, vary in the magnitude or character of the resulting epigenetic responses. Nonetheless, it is important to note that, apart from supraphysiological doses of fructose (> 75 g) consumed in a bolus, fructose consumed as part of a typical diet is likely effectively metabolized by the small intestine, or converted into glucose, which will then be metabolized the same way as glucose in the diet (as simple sugar, or a digestive product of starch), and organic acids [50, 51]. Apart from rate of absorption, glucose from sucrose or HFCS does not behave in any way differently from glucose from other carbohydrates in the metabolic pathway. Therefore, the epigenetic mechanism does not appear to be a plausible explanation.

To balance the academic debate on the effects of on metabolic health, it is essential to consider the potential lack of adverse metabolic effects of free sugars (except those from SSBs) at physiological doses [6]. The human body is well-equipped to handle the metabolism of moderate amounts of free sugars, particularly when consumed as part of a balanced diet. When free sugars are consumed in solid food matrices, the physiological response may be different, as the slower digestion and absorption rate, as well as the presence of other macronutrients and fibre, can modulate the metabolic impact [36]. This suggests that free sugars from solid food sources may not have the same detrimental effects as those from SSBs.

## Epidemiological Evidence

A substantial body of epidemiological research has examined the relationship between sugar consumption and T2DM. As discussed previously, recent umbrella reviews and systematic meta-analyses demonstrate that total sugar or fructose intake shows no clear association with T2DM risk, with significant heterogeneity observed across individual studies attributable to variations in dietary assessment methods, follow-up duration, and outcome ascertainment [28–30]. This lack of consistent association at the aggregate level masks important heterogeneity based on food source, as revealed by more granular analysis of specific dietary sources of fructose-containing sugars. In one meta-analysis [79], SSBs, fruit drinks and unspecified fruit juice showed increased risk. In contrast, yogurt and chocolate were associated with lower T2DM risk despite containing sugars. Importantly, 100% fruit juice showed no significant association with T2DM risk, and dose-response analysis revealed a U-shaped relationship for whole fruit consumption, with protective effects observed up to approximately four servings per day. No associations were found for breakfast cereal, honey, jam spreads, cookies and cakes, desserts, or sweetened tea and coffee.

The pattern emerging from these food source-specific analyses demonstrates that delivery form and food matrix fundamentally modify the relationship between sugar intake and T2DM risk. The comprehensive umbrella review by Banjarnahor, et al. [28] and other meta-analyses have reinforced the robustness of this particular association, generally reporting risk estimates ranging from 13% to 30% increased T2DM incidence associated with habitual SSB consumption [31–33, 80–84]. These findings demonstrate remarkable consistency across diverse populations and study designs, leading to widespread recognition of the SSB-T2DM relationship. This convergence of evidence establishes SSB consumption as a key modifiable dietary factor in T2DM prevention.

The distinction between different juice types warrants particular attention given their intermediate position between whole fruits and SSBs [31–33, 80, 85]. While often perceived as healthier alternatives to SSBs, fruit juices can contain similar amounts of free sugars. The epidemiological evidence reveals important nuances in these associations. For fruit juice generally, a meta-analysis reported 5% higher risk for T2DM per one serving/day in a meta-analysis, but no association was found when only studies that ascertained T2DM objectively were considered [31]. Another meta-analysis conducted sub-group analysis and found that the association between fruit juice intake was found (RR 1.28, 95%CI 1.04–1.59) but only if the fruit juice was sweetened, but not in 100% fruit juice [84]. This pattern aligns with the Khan, et al. ^80^ findings showing no association for 100% fruit juice while unspecified fruit juice showed modest increased risk. It must be pointed out that the quality of fruit juice studies is mixed due to the lack of clear definition of 100% fruit juice [86]. The differential associations observed for sweetened vs. 100% fruit juice suggest that added sugars beyond those naturally present in fruit may be the critical factor, though methodological limitations in juice classification warrant cautious interpretation.

Most research has investigated how free sugars consumption impacts T2DM risk. However, some studies explore the possibility of reverse causation - the possibility that T2DM may influence sugar intake preferences and behaviours. Reverse causation would also tend to attenuate rather than strengthen observed associations, as individuals concerned about weight gain or diabetes risk typically reduce their intake of free sugars, particularly from obvious sources like SSBs. This same mechanism is commonly invoked to explain the paradoxical positive associations observed between non-nutritive sweetener use and T2DM risk. Therefore, reverse causation is unlikely to explain the positive associations observed for SSBs, and may actually lead to underestimation of true effects.

Genetic studies have provided valuable insights in this regard. Gene-diet interaction studies suggest that higher SSB consumption facilitates the phenotypic expression of genetic predisposition to obesity, with stronger genetic associations observed among high SSB consumers compared to low consumers [87]. This indicates that liquid free sugar intake may amplify genetic risk rather than representing an independent risk factor. In contrast, some studies have shown that genetic variants linked to sweet preference or metabolic traits may be associated with both higher SSB intake and elevated T2DM risk, supporting a potential genetic contribution to both behaviour and disease risk [88]. This highlights the complex interplay between genetics, dietary habits and underlying metabolic health.

## Confounding Factors and Limitations

Several factors complicate the interpretation of the relationship between free sugars and T2DM. Free sugars consumption is often associated with higher total energy intake and obesity, both independent risk factors for T2DM [89]. It is also important to point out that foods high in free sugars, with the exception of SSB, can also be high in saturated fats and sodium (e.g. in cakes and pastries), hence attributing the relationships of T2DM to free sugars alone may be problematic and misleading. While these statistical challenges complicate etiological inference, from a public health policy standpoint, the co-occurrence of multiple nutrients of concern in SSBs and ultra-processed foods may actually facilitate more effective food-based guidance and intervention strategies. There is also evidence that higher sugar intake, especially free sugars from SSB, was associated with poorer overall diet quality [90–92], which, in turn, is a strong predictor of health [93]. Disentangling these potential confounding effects from the direct impact of free sugars on T2DM is challenging, and as suggested above, there appears to be a lack of biological foundations at typical dietary doses. Physical activity levels can also modify the association, as individuals with high sugar intake but also high physical activity may have lower risk compared to sedentary individuals. Genetic factors further influence the susceptibility of an individual to T2DM [94–96] and may interact with dietary factors [97, 98], including free sugars intake.

Accurate assessment of free sugar intake in epidemiological studies is another known methodological limitation. There are many challenges in accurately estimating dietary sugar intake as dietary assessment methods have their own limitations [99, 100]. Some studies that explored the relationships between sugar intake and T2DM cited in this review involved secondary data analysis, where the original studies were not aimed to accurately assess dietary sugar intake. This creates several methodological challenges. First, the selected dietary assessment methods may not have been optimized for precise sugar intake measurement. Second, there is a significant gap in accurate data regarding the free sugars content of different foods, particularly given the rapidly expanding array of new food products [4]. Third, operationalizing the free sugars definition presents practical challenges in classifying foods along the spectrum from intact to processed. The boundary between intact fruits and vegetables vs. processed sources becomes ambiguous in foods such as soups, mixed dishes, purees, and minimally processed items, creating inconsistencies in how different research teams categorize and quantify free sugar content [5]. Researchers often estimate free sugar intake by using the distinction between core and discretionary foods as a proxy due to the lack of item-specific free sugar data [5, 101, 102]. However, this method presents challenges, particularly in differentiating sugars naturally present in core food groups like fruits and dairy from those added in discretionary products. The difficulty is heightened with hybrid items, such as fruit cake, which blend core food ingredients with discretionary components, complicating their classification and analysis. Additionally, potential underreporting of dietary intake by participants, especially those with T2DM, further complicates the accuracy of these assessments [103]. As dietary methods are subjective (prone to bias) and often rely on memory, under-reporting is common and remains a major challenge in accurate quantification of dietary intake [99, 104]. These measurement limitations would generally attenuate observed associations, potentially explaining weak epidemiological signals. However, it is worth noting that SSBs may be less subject to measurement error than solid sugar-containing foods, as they are consumed in discrete, standardized units that are easier to recall accurately. If this differential measurement error exists, it might partially explain why stronger associations are observed for SSBs compared to free sugars from solid food sources, beyond any true mechanistic differences. Nonetheless, direct evidence for this measurement advantage of beverages is limited. Attempts have been made to address this challenge, including the use of objective measurements that do not rely on memory. Two such methods are the potential use of carbon isotope ratio [105, 106] and urinary sucrose/fructose excretion [107] as predictors of sugar intake. To-date, findings from validation studies indicate that these methods are far from optimal in predicting added/free sugar intake (for details please see our Letter to Editor [108] and perspective paper [107]).

It is also important to consider the ecological validity of the studies on sugar and its potential role in T2DM development, e.g., cross-sectional studies that are susceptible to reverse-causation. Extrapolating findings from studies using supraphysiological sugar doses (often > 50% of total energy as free sugars in experimental protocols, a level rarely seen in < 5% of the population) to real-world dietary patterns may lead to overstated conclusions about the metabolic impact of sugar at typical consumption levels [6, 25, 109].

## Public Health Implications

Many national and international organizations have revised dietary guidelines to recommend limiting free sugars intake [4]. It is important to recognize that public health guidance on sugar reduction addresses multiple health outcomes beyond T2DM. Dental caries represents a major consideration, with the WHO’s quantitative recommendation to limit free sugars to less than 10% of total energy intake based primarily on dental health evidence [2]. Additional rationales include diet quality improvement, prevention of excessive energy intake and overweight, and reduction of other cardiometabolic risks. The WHO recommends reducing free sugars intake to less than 10% of total energy intake, with a further reduction to below 5% for additional health benefits [2]. This has prompted numerous public health initiatives and policy interventions aimed at reducing population-level sugar intake. For example, several jurisdictions, including Mexico, Portugal., and various cities in the U.S., have introduced excise taxes on SSBs [110]. These “soda taxes” have been shown to effectively reduce SSB consumption and have the potential to generate revenue that can be reinvested in public health programs [111]. Complementary strategies, such as subsidies for healthier foods and beverages, have also been explored to encourage dietary shifts away from unhealthy foods and beverages, including those high in free sugars [112, 113].

Consistent with the evidence reviewed here, SSBs have appropriately become a major focus of sugar reduction efforts in public health policy. However, this emphasis on liquid sugar sources must be balanced with clear messaging that promotes consumption of nutrient-dense whole foods containing naturally occurring sugars. An unintended consequence of broad “reduce sugar” messaging has been the perpetuation of misconceptions about certain fruits being “too high in sugar” for regular consumption. Fruits such as bananas, grapes, and watermelon have been inappropriately stigmatized in popular discourse despite their nutrient density and consistent association with positive health outcomes [114, 115]. Public health communication should explicitly affirm that all fruits are recommended regardless of sugar content, as the fiber, vitamins, minerals, and phytochemicals they provide confer health benefits that far outweigh concerns about their natural sugar content. Clear differentiation between free sugars from SSBs and intrinsic sugars in whole fruits is essential to prevent well-intentioned sugar reduction efforts from inadvertently discouraging consumption of foods that should form the foundation of healthy dietary patterns.

Improved food labelling, particularly regarding added/free sugars content, and consumer education initiatives are being implemented to help individuals make informed dietary choices. For example, the U.S. Food and Drug Administration has mandated the inclusion of added sugars content on Nutrition Facts labels [116, 117], which provides consumers with clear, actionable information about the sugar content in their food choices. Beyond improved transparency through labelling, there is growing demand for product reformulation across the food industry. This shift is driven by both consumer interests and public health initiatives. Several countries have established structured approaches to sugar reduction, implementing either voluntary industry targets or mandatory reduction requirements for processed foods [118, 119]. This creates measurable benchmarks for manufacturers while allowing for gradual adaptation of product formulations to maintain consumer acceptance.

However, despite these efforts, the prevalence of T2DM continues to rise globally [1]. Several factors may contribute to the failure of current public health strategies to effectively address the issue. First, the complexity of the relationship between free sugars consumption and T2DM risk, as well as the potential lack of adverse metabolic effects of free sugars (except those from SSBs) at physiological doses, may have limited the impact of these strategies. The focus on a blanket reduction of all free sugars, without distinguishing the differential metabolic effects of various sources, may have led to overly simplistic recommendations that fail to account for the nuances of the underlying science [4]. Second, the implementation and enforcement of these strategies have been uneven across different regions and populations. The effectiveness of policy interventions, such as sugar taxation, has been variable and often limited by industry workarounds and consumer resistance [120–123]. Third, the prevailing emphasis on individual behavioural change, through measures like consumer education and food labelling, may have underestimated the broader societal and environmental factors that shape dietary patterns and sugar consumption. The food environment, including the ubiquity of cheap, highly palatable, and energy-dense processed foods, can overwhelm individual efforts to make healthier choices [124]. Finally, the lack of comprehensive, concerted, multi-stakeholder approaches that engage policymakers, the food industry, healthcare providers, and communities has limited the effectiveness of current strategies. A more coordinated, systems-level approach that addresses the social, economic, and environmental determinants of sugar consumption may be necessary to achieve meaningful and sustained reductions in T2DM prevalence.

Public health interventions could enhance their effectiveness by adopting a more targeted approach. While many jurisdictions have already prioritized SSB reduction, as evidenced by taxation policies, front-of-package labelling initiatives, and focused public health campaigns, this approach warrants more universal and explicit adoption in dietary guidance globally, as SSBs demonstrate the most consistent association with adverse metabolic outcomes [4, 6, 109, 125]. Where SSB reduction strategies are already established, efforts should focus on strengthening implementation and ensuring consistent messaging across all public health channels. This focused strategy should be complemented by educational initiatives that clearly communicate the distinct metabolic effects of free sugars from various dietary sources. Such education should emphasize that moderate consumption of free sugars from solid foods, especially fruits and vegetables, typically poses minimal health risks, helping consumers make more nuanced and informed dietary decisions.

## Future Research Directions

Future research in two major areas is needed. First, fundamental mechanistic research is needed to advance our understanding of how sugars influence health. For example, research into how genetic factors and individual metabolic profiles modify the effects of free sugars on T2DM risk could help identify high-risk populations and inform personalized dietary recommendations and targeted interventions. Given the limited mechanistic plausibility of microbiota as primary mediators of free sugar effects at typical intake levels, research priorities should focus on direct metabolic pathways rather than microbiome-mediated mechanisms. However, if microbiome studies are conducted, they must be carefully designed to account for the complexity of human diets. The manipulation of a specific dietary factor (e.g. sugars or SSB) can alter the overall dietary intake and/or diet quality, which can have unintended consequences on gut microbiome outcomes [126]. Finally, investigation of how free sugars consumption may influence epigenetic modifications and subsequent T2DM risk could reveal new mechanistic pathways and potential interventions. Although these mechanistic studies may not have immediate public health applications, they may inform personalized nutrition interventions in the future.

The second area of research will have more direct implications on public health nutrition. For example, research that differentiates the metabolic effects of free sugars from various sources, e.g. SSBs, solid foods, and natural sources, will refine our understanding of the underlying mechanisms and tailor food-specific public health strategies accordingly. Research that compares the physiological responses to typical dietary vs. supraphysiological doses of fructose and other free sugars will also be crucial in formulating relevant public health messaging on sugar intake. While challenging to conduct, long-term RCTs are needed to establish causality and quantify the direct effects of free sugars reduction on T2DM risk. Such studies would provide stronger evidence to guide public health policies and clinical recommendations.

The development of a comprehensive food, recipe and nutrient database will be imperative to better estimate free sugar intake would allow future research that focus on:

1. *Dose-response relationships*: More precise quantification of the relationship between free sugars intake and T2DM risk at different levels of consumption, particularly within the range of typical dietary intake (35–50 g/day free sugars, with specific attention to intakes between 50 and 125 g/day representing the transition from median to high consumption). Since moderate consumption of free sugars from diverse sources within a balanced diet is u.
2. *Long-term effects*: Studies examining the cumulative effects of moderate free sugars consumption (35–50 g/day) over extended periods, as opposed to short-term interventions with high doses (> 125 g/day or > 25% of total energy).
3. *Interaction with other dietary components*: Investigation of how the metabolic effects of free sugars are modulated by other nutrients, dietary patterns, and overall diet quality.
4. *Mechanisms of SSB-specific effects*: Further elucidation of why SSBs appear to have more pronounced metabolic effects compared to other sources of free sugars.
5. *Population-specific responses*: Examination of how different populations (e.g., based on age, ethnicity, or metabolic health status) respond to free sugars consumption.
6. *Effectiveness and harmonisation of public health interventions*: Evaluation of the long-term impact of various policy measures aimed at reducing free sugars intake on T2DM incidence and prevalence.
7. *Alternative sweeteners*: Investigation of the metabolic effects of non-nutritive sweeteners (to replace free sugars) and their potential role in T2DM prevention or management.
8. *Timing of sugar consumption*: Exploration of whether the timing of free sugars intake (e.g., with meals vs. between meals) influences their metabolic effects.

## Discussion

This narrative review synthesizes the current evidence on the association between free sugars consumption and T2DM risk. While most epidemiological studies support a positive association, particularly for SSBs, the relationship is complex and influenced by various confounding factors. Proposed mechanisms include effects on insulin resistance, *β-*cell function, oxidative stress, inflammation, and hepatic lipid metabolism. While gut microbiota, and epigenetic modifications have also been proposed, these are less mechanistically plausible given the rapid proximal absorption of free sugars at typical intake levels. However, the human body can metabolize moderate amounts of free sugars at typical dietary levels without triggering significant insulin resistance or *β*-cell dysfunction. Similarly, oxidative stress, and inflammation associated with typical free sugar intake likely remain within the body’s homeostatic capacity.

Sugars also enhance the palatability of foods and beverages, influencing dietary choices and habits. This can lead to overconsumption of sugary foods, excessive energy and fat intake, displacement of healthier foods, and long-term positive energy balance, which contribute to obesity and poorer diet quality - key risk factors for T2DM. These behavioural impacts complicate the understanding of the role of sugars in T2DM development.

The limited impact of public health measures aimed at reducing free sugar consumption on T2DM prevalence highlights the need for more targeted strategies. A primary challenge lies in translating complex scientific evidence into clear, actionable public health messaging [4, 125]. While expert consensus has converged on several key points, particularly regarding the relationship between SSBs and multiple health outcomes including T2DM, dental caries, and diet quality, communicating these nuances to the public without oversimplification remains difficult. The message that “all sugars are harmful” is simpler to convey than differentiated guidance based on delivery form and dietary context, yet overly broad messaging may inadvertently discourage consumption of nutrient-dense foods like fruits. Beyond communication challenges, substantive barriers include inconsistent policy implementation across jurisdictions, the difficulty of achieving meaningful changes in the food environment and consumer behaviour, and inadequate coordination among government agencies, health organizations, and the food industry.

Our review suggests that more explicit differentiation in dietary guidance between SSB-sourced sugars and those from whole foods could improve message clarity and support more appropriate dietary choices. However, we acknowledge that current broad recommendations to limit free sugars serve multiple important public health objectives. Dental caries prevention, maintenance of diet quality, and prevention of excessive energy intake all provide valid rationales for comprehensive sugar reduction guidance that extends beyond metabolic considerations alone. Future research examining individual variation in metabolic responses to different sugar sources and delivery forms could inform more personalized implementation strategies, though population-level policies targeting the food environment must remain the primary focus of public health efforts. The distinction we propose is not between population vs. individual approaches, but rather between undifferentiated sugar reduction messaging and evidence-based guidance that explicitly accounts for the differential health impacts of various sugar sources while acknowledging the full range of considerations that inform dietary recommendations.

To address these challenges, long-term RCTs and studies investigating the metabolic effects of free sugars from different sources are crucial for improving understanding and refining prevention efforts. In both clinical practice and public health, it is important to consider the full scope of available evidence while recognizing its limitations. These efforts are essential for refining prevention strategies and enhancing clarity in both clinical practice and public health. While evidence supports reducing free sugar intake, especially from SSBs, recommendations should be adaptable to individual needs and preferences, ensuring a balanced approach to dietary guidance. Since moderate consumption of free sugars (35–50 g/day, representing population median intake, or < 10% of total energy intake as recommended by WHO) from diverse sources within a balanced diet is unlikely to have significant metabolic consequences for most individuals [8], public health messages and interventions should reflect this nuance by focusing on reducing SSB consumption and promoting overall dietary quality, rather than demonizing all free sugar sources [4, 125].

## Conclusion

The relationship between free sugars and T2DM is complex and multifaceted. While caution is warranted regarding high intake of free sugars (> 125 g/day or > 25% of total energy), particularly from SSBs, moderate consumption (35–50 g/day) as part of a balanced diet is unlikely to significantly increase T2DM risk for most individuals. Future research and public health strategies should focus on refining our understanding of the differential effects of various sugar sources and developing targeted interventions that address the broader determinants of dietary patterns and metabolic health.

## Key References

- Yan, R. R., Chan, C. B. & Louie, J. C. Y. Current WHO recommendation to reduce free sugar intake from all sources to below 10% of daily energy intake for supporting overall health is not well supported by available evidence. Am. J. Clin. Nutr. 116, 15–39 (2022). https://doi.org/10.1093/ajcn/nqac084.
  - ⚬ This paper rigorously challenges the strength of the evidence behind the WHO's current recommendations on sugar intake. It highlights how most research linking sugar to poor health relies heavily on studies of sugar-sweetened beverages, with much weaker or inconsistent findings for sugars in solid foods. The authors argue that these limitations make current public health guidelines potentially misleading and in need of revision.
- Gillespie, K. M., Kemps, E., White, M. J. & Bartlett, S. E. The Impact of Free Sugar on Human Health-A Narrative Review. Nutrients 15, 889 (2023). https://doi.org/10.3390/nu15040889.
  - ⚬ This article critically synthesizes decades of research to clarify the debated role of free and added sugars in major health conditions, including obesity, diabetes, cardiovascular disease, and cognitive decline. It distinguishes between types of sugars and carbohydrates, identifies methodological flaws in earlier studies, and brings forward evidence that supports current dietary guidelines.
- Turck, D. et al. Tolerable upper intake level for dietary sugars. EFSA J 20, e07074 (2022). https://doi.org/10.2903/j.efsa.2022.7074.
  - ⚬ This EFSA opinion provides critical scientific backing for reconsidering current sugar guidelines. The panel found only moderate evidence linking added sugars to obesity and dyslipidemia, with lower certainty for other health outcomes, supporting calls for more nuanced approaches to sugar policy that move beyond arbitrary quantitative limits.
- Cara, K. C. et al. Associations between Intake of Dietary Sugars and Diet Quality: A Systematic Review of Recent Literature. Nutrients 16 (11), 1549 (2024). https://doi.org/10.3390/nu16111549.
  - ⚬ This article systematically reviews and synthesizes recent studies on how different types of dietary sugars relate to overall diet quality, identifying consistent negative associations between added or free sugar intake and essential nutrients. It fills a major gap left by outdated or methodologically weak reviews, applies rigorous selection and bias assessment criteria, and highlights the limitations of current research, pointing clearly to the need for higher-quality prospective studies.
- Louie, J. C. Y. The time has come to reconsider the quantitative sugar guidelines and related policies. npj Science of Food 8, 88 (2024). https://doi.org/10.1038/s41538-024-00332-4.
  - ⚬ This commentary challenges the scientific foundation of current sugar intake guidelines, arguing that the WHO's widely-cited recommendations to limit added/free sugars to less than 10% of total energy intake are based on only "moderate" quality evidence for dental health and "very low" quality evidence for broader health benefits. It argues that strict quantitative sugar limits that may be impractical and potentially counterproductive.

## Data Availability

No datasets were generated or analysed during the current study.

## Declarations

### Declarations of use of generative AI

During the writing of this review, the authors used Claude 4.5 sonnet to improve the grammar, flow and coherence of the text they drafted. The corresponding author then checked and confirmed the accuracy of the revised text, and takes primary responsibility for the content of the final article presented.
